# Chromosomal painting and ZW sex chromosomes differentiation in *Characidium *(Characiformes, Crenuchidae)

**DOI:** 10.1186/1471-2156-12-65

**Published:** 2011-07-25

**Authors:** Tatiana C Machado, José C Pansonato-Alves, Marcela B Pucci, Viviane Nogaroto, Mara C Almeida, Claudio Oliveira, Fausto Foresti, Luiz AC Bertollo, Orlando Moreira-Filho, Roberto F Artoni, Marcelo R Vicari

**Affiliations:** 1 Departamento de Biologia Estrutural, Molecular e Genética, Universidade Estadual de Ponta Grossa, Av. Carlos Cavalcanti, 4748, Ponta Grossa-PR, 84030-900, Brazil; 2 Departamento de Morfologia, Universidade Estadual Paulista, Distrito de Rubião Junior, s/n, Botucatu-SP, 18618-970, Brazil; 3 Departamento de Genética e Evolução, Universidade Federal de São Carlos, Rodovia Washington Luís Km 235, São Carlos-SP, 13565-905, Brazil

## Abstract

**Background:**

The *Characidium *(a Neotropical fish group) have a conserved diploid number (2n = 50), but show remarkable differences among species and populations in relation to sex chromosome systems and location of nucleolus organizer regions (NOR). In this study, we isolated a W-specific probe for the *Characidium *and characterized six *Characidium *species/populations using cytogenetic procedures. We analyzed the origin and differentiation of sex and NOR-bearing chromosomes by chromosome painting in populations of *Characidium *to reveal their evolution, phylogeny, and biogeography.

**Results:**

A W-specific probe for efficient chromosome painting was isolated by microdissection and degenerate oligonucleotide primed-polymerase chain reaction (DOP-PCR) amplification of W chromosomes from *C. gomesi*. The W probe generated weak signals dispersed on the proto sex chromosomes in *C. zebra*, dispersed signals in both W and Z chromosomes in *C. lauroi *and, in *C. gomesi *populations revealed a proximal site on the long arms of the Z chromosome and the entire W chromosome. All populations showed small terminal W probe sites in some autosomes. The 18S rDNA revealed distinctive patterns for each analyzed species/population with regard to proto sex chromosome, sex chromosome pair, and autosome location.

**Conclusions:**

The results from dual-color fluorescence *in situ *hybridization (dual-color FISH) using W and 18S rDNA probes allowed us to infer the putative evolutionary pathways for the differentiation of sex chromosomes and NORs, from structural rearrangements in a sex proto-chromosome, followed by gene erosion and heterochromatin amplification, morphological differentiation of the sex chromosomal pair, and NOR transposition, giving rise to the distinctive patterns observed among species/populations of *Characidium*. Biogeographic isolation and differentiation of sex chromosomes seem to have played a major role in the speciation process in this group of fish.

## Background

The Crenuchidae is widespread in freshwater systems of the South and Central Americas [1]. The *Characidium *is the most representative group in this family, comprising 50 valid species [1]. This is a particularly interesting Neotropical fish group for cytogenetic studies because it presents a diversified model of sex chromosomes [2]. In the *Characidium*, a diploid number of 50 chromosomes is observed in all studied species and a karyotype of 32 m + 18 sm is most commonly reported [2-10].

Although the diploid number is conserved, the *Characidium *exhibit remarkable interspecific and interpopulation differences, such as (*i*) inter- and intraindividual variation of B chromosomes in some species [3-5]; (*ii*) a sex chromosome system with ZZ/ZW female heterogamety [2-9]; (*iii*) variation in the location and number of rDNA sites in distinct chromosomal pairs [2-4,8,10]; and (*iv*) occurrence of natural triploidy in *C. gomesi *and *C*. cf. *zebra *[6,10].

The first cytogenetic report in *Characidium *was for *C. zebra *[11] and, so far, this is the only species where heteromorphic sex chromosomes are absent [4,6,10]. Based on morphological analysis, *C. zebra *exhibits several plesiomorphic traits, being placed as basal in *Characidium *phylogeny [12]. The chromosomal characters of *C. zebra *have already been referred to as putatively plesiomorphic for the genus [2]. Therefore, changes in the karyotypes observed in other species of *Characidium*, when compared with *C. zebra*, should be the result of secondary chromosomal rearrangements.

The karyotypes of *C. lauroi *and *C. alipioi *have been described, and while the latter was characterized by the presence of a ZZ/ZW sex chromosome system, *C. lauroi *was considered homomorphic because of the lack of differential heterochromatic bands [7]. However, further comparative studies in seven species of this genus (*C. zebra*, *C. lanei*, *C. pterostictum*, *C. lauroi*, *C. oiticicai*, *Characidium *sp. and *C. schubarti*) showed that only *C. zebra *lacked differentiated sex chromosomes and the other species presented sex chromosomes in different W-heterochromatin amplification stages and had terminal NORs on both Z and W chromosomes [4]. *Characidium gomesi *is another species with highly differentiated sex chromosomes, bearing entirely heterochromatic W chromosomes and NORs on autosomes [2,3,6].

A sex proto-chromosome pair for *Characidium *species was proposed, from which both Z and W chromosomes have evolved by structural rearrangements, such as duplications, deletions, and/or inversions. By contrast, the heterochromatin amplification of W was regarded as a predominant event in the differentiation of the sex chromosomal pair [2]. Later, it was inferred that the NORs on ZW chromosomes in most species of *Characidium *represented an ancestor state of sex proto-chromosomes, prior to heterochromatin amplification [4]. Heterochromatin amplification in one of the sex chromosomes driven by the accumulation of certain repetitive DNA classes in one homologous is relatively common in fish [13]. Such a process would favor recombination suppression and thereby the independent evolution of chromosomes or chromosomal regions [14-17].

In the present study, we analyzed the origin, composition, and differentiation of sex and NOR-bearing chromosomes in populations of *C. zebra*, *C. lauroi*, and *C. gomesi*, using chromosomal painting with a W-specific probe from *C. gomesi*. We also mapped the 18S rDNA to infer the evolution, phylogeny and biogeography of the group.

## Results

**Figure 1:**
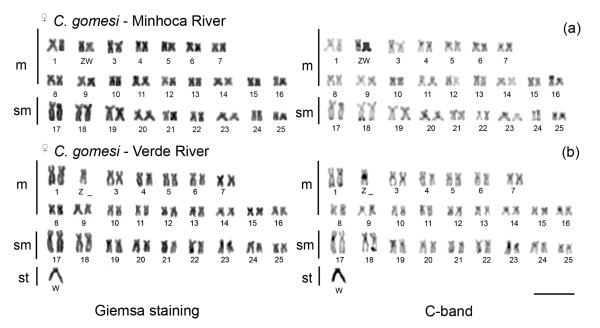
**Karyotypes of females of *C. gomesi***. In (**a**) population from the Minhoca stream, São Francisco River basin (MG). The W chromosome is entirely heterochromatic and easily identifiable by C-banding, used in the chromosomal microdissection and obtaining of W-specific probe. In (**b**) population from the Verde River, Tibagi River basin (PR). Bar = 10 μm.

Populations of *C. zebra *from Passa Cinco River, Upper Paraná basin (SP), *C. lauroi *from Grande stream, Paraíba do Sul basin (SP), and *C. gomesi *from Grande River, Upper Paraná basin (SP), Alambari stream, Tietê/Upper Paraná basin (SP), Minhoca stream, São Francisco River basin (MG), and Verde River, Tibagi River/Upper Paraná basin (PR) have a conserved diploid number of 50 chromosomes, most of which are metacentric/submetacentric (m/sm) (table 1). The populations of *C. gomesi *from Minhoca stream-MG and Verde River-PR are analyzed in the present work for the first time, and showed a differentiated ZZ/ZW system (Figure 1 and table 1). In both populations, the Z chromosome is a medium-sized m chromosome carrying a proximal heterochromatin band on the long arms. The W chromosome is an entirely heterochromatic m, smaller than Z chromosome in specimens from Minhoca stream (Figure 1c), whereas the W chromosome in the population from Verde River is a heterochromatic subtelocentric (st) chromosome, slightly larger than Z (Figure 1d). Therefore, the karyotype formulae differ between both populations because of the morphological differences in the W chromosome (Figure 1a, c and table 1).

**Table 1:** Chromosomal formula, sex chromosome system occurrence, W-specific probe sites and 18S rDNA sites on the chromosomes of the *Characidium *species/populations analysed.

Species	River	Chromosomal Formula	Sex System	W probe sites	18S rDNA sites/localization	Figure 3 relation
*C. zebra*	Passa Cinco	♂ ♀ 32 m+18 sm	Absent	Dispersed in autosomes	23 pair/subterminal	(a)
*C. lauroi*	Grande stream	♂ 32 m+18 sm ♀ 31 m+18 sm+1st	ZZ/ZW	Autosomal and dispersed in the W chromosome	Z and W/terminal	(b)
*C. gomesi*	Alambari	♂ ♀ 32 m+18 sm	ZZ/ZW	Z in proximal region of the long arm, entire m W chromosome and some terminal minor sites over autosomes	Z and W/terminal and 19 pair/terminal in just one homologue in males	(c and d)
*C. gomesi*	Grande	♂ ♀ 32 m+18 sm	ZZ/ZW	Z in proximal region of the long arm, entire m W chromosome and some terminal minor sites over autosomes	17 pair/terminal	(e and f)
*C. gomesi*	Minhoca stream	♂ ♀ 32 m+18 sm	ZZ/ZW	Z in proximal region of the long arm, entire m W chromosome and some terminal minor sites over autosomes	17 pair/terminal	(g)
*C. gomesi*	Verde	♂ 32 m+18 sm ♀31 m+18 sm+1st	ZZ/ZW	Z in proximal region of the long arm, entire st W chromosome and some terminal minor sites over autosomes	17 and 22 pairs/terminal and just one homologue of pairs 1 and 20/terminal	(h)

**Figure 2:**
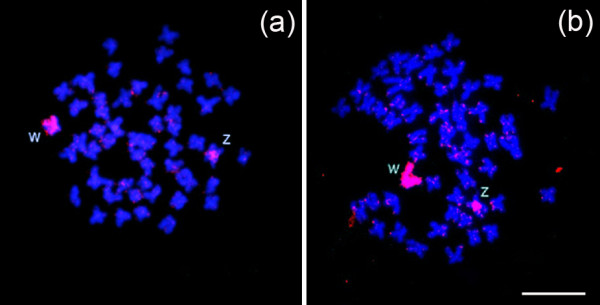
**W *Characidim *probe efficiency**. In (a) female metaphases of *C. gomesi *from the Minhoca stream population and (b) from the population of Verde River (PR) showing the chromosomes hybridised with the W-specific probe. Bar = 10 μm.

DOP-PCR amplification of C-banded W chromosomes from *C. gomesi *and hybridization of W probe onto the metaphasic chromosomes of *C. gomesi *from Minhoca stream and Verde River was highly efficient, revealing positive signals at the terminal regions of some autosomes, at the proximal region on the long arms of the Z chromosome, and the entire W chromosome (Figure 2a and 2b).

**Figure 3:**
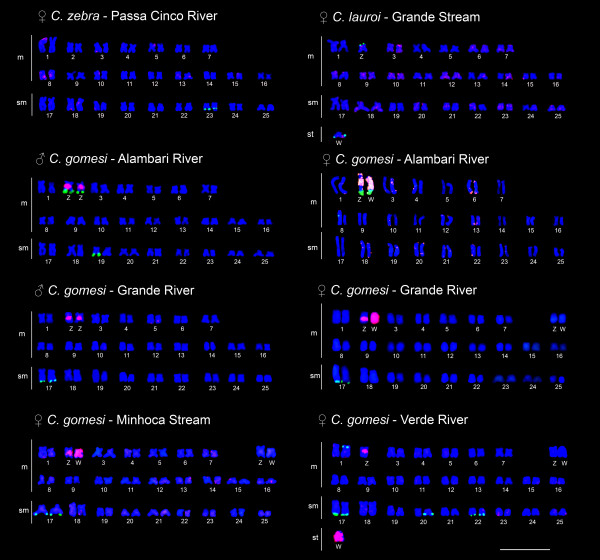
**Location of signals after hybridization with W-specific (red) and 18S rDNA (green) probes in the karyotypes of species/populations of *Characidium *stained with DAPI**. Bar = 10 μm.

Dual-color FISH using 18S rDNA and W probes revealed distinctive patterns for each analyzed population. Thus, *C. zebra*, presented small sites related to the W probe dispersed through the karyotype and an 18S rDNA site in the sm pair 23 (Figure 3a and table 1). *Characidium lauroi *presented 18S rDNA signals on the heteromorphic m pair 2, regarded as the sex chromosomes in this species, and hybridization with the W probe revealed weak signals dispersed on both the W and Z chromosomes, in addition to some terminal autosomal regions (Figure 3b and table 1). The population of *C. gomesi *from the Alambari stream showed 18S rDNA sites in the Z and W chromosomes, besides an additional terminal site in homologous pair 19, exclusively for males (Figure 3c, d and table 1). The W probe hybridized mainly on the proximal region of the Z chromosome and to almost the entire m W chromosome, except for the NORs, besides on some minor terminal sites in the autosomes (Figure 3c, d and table 1). *Characidium gomesi *from the Grande River and Minhoca stream presented similar 18S rDNA signals in the sm pair 17. The W probe hybridized mainly on the proximal region of the Z chromosome and the entire m W chromosome, besides some small terminal sites on the autosomes (Figure 3e, f, g and table 1). On the other hand, *C. gomesi *from the Verde River showed six positive 18S rDNA sites. FISH with the W probe revealed small terminal sites in some autosomes, besides a proximal site on the long arms of the Z chromosome and the entire st W chromosome (Figure 3h, table 1).

## Discussion

### The karyotypic analysis

The populations of *C. gomesi *(Minhoca stream and Verde River-analyzed for the first time in the present work) are highly similar (2n = 50 chromosomes, moistly m/sm) to other previously studied populations. Furthermore, although 32 m + 18 sm represents the most common karyotype in this group, chromosomal formulae variations have already been reported, indicating that some rearrangements, such as pericentric inversions, might have taken place in the chromosomal diversification of this genus [2,4,5]. Nonetheless, in spite of the apparent karyotype conservation, the populations of *C. gomesi *have diversified mainly in the structure and morphology of their Z and W chromosomes and the number of major rDNA sites [2]. Amongst the several analyzed populations of *C. gomesi*, only one showed NORs located on sex chromosomes [5]. Therefore, among the *Characidium *species bearing heteromorphic sex chromosomes, only *C. alipioi *and *C. gomesi *(excepting the sample from the Alambari stream) have NORs exclusively on autosomes, suggesting this is a derived feature [4,5].

In general, it is hypothesized that sex chromosomes evolved from a specific pair of autosomes carrying some sex-determining gene(s). Subsequently, the newly formed sex chromosomes stopped recombination in a small region around the sex-determining locus [18]. Sex chromosomes in this early stage of evolution are not cytologically distinguishable (homomorphic). The process of recombination suppression then progressed through almost the entire sex chromosome. The exchange restraint between homologs during meiosis could be caused by structural chromosomal rearrangements [19] or by the accumulation of certain repetitive DNA classes in one homolog in the pair of sex proto-chromosomes [20,21]. The results obtained permit speculation concerning this sex chromosome differentiation.

### Sex chromosomes and NOR differentiation in *Characidium*

Comparative chromosomal painting using the W-specific probe of *C. gomesi *demonstrated that the repetitive DNAs present on sex chromosomes from different populations of these species are highly conserved, despite the morphological diversification of these chromosomes. In addition, the occurrence of W-specific sites on autosomes, albeit dispersed over terminal regions in all analyzed populations/species, reveals that the repetitive units that compose and differentiate the sex chromosomes in *Characidium *are widespread in the genus. This corroborates the hypothetical common origin of the ZZ/ZW system [2,5] and its monophyletic status, giving rise to hypothesis of serial transformation of these chromosomes within the genus *Characidium*.

**Figure 4:**
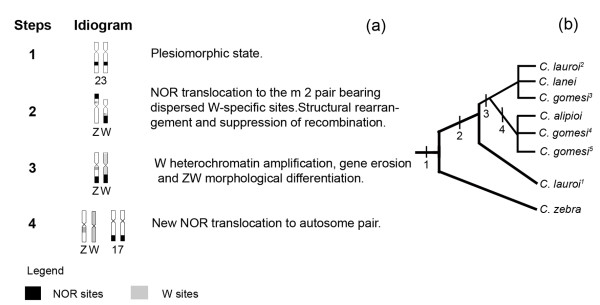
**Stages in the differentiation of sex and NOR-bearing chromosomes among species and populations of *Characidium***. In (a) steps and idiogram representation of the NOR and W sequences rearrangements; (b) hypothesised relationship among species analysed. ^1^*C. lauroi *of the Ribeirão Grande population; ^2^*C. lauroi *of the Ubatuba population; ^3^*C. gomesi *of the Alambari population; ^4^*C. gomesi *of the Minhoca population and; ^5^*C. gomesi *of the Verde population.

Based on this scenario, it is possible to hypothesize that the sex chromosomes of *Characidium *had a common origin and diversified in sequential stages (Figure 4). Therefore, the ancestor condition, without morphologically differentiated sex chromosomes, seems to be present in *C. zebra*, with NORs located at the subterminal region of a small sm pair (23^rd^) and W-specific regions dispersed at the terminal regions of some chromosomes (Figure 4, step 1).

The next step probably involved translocation of the NOR to the 2^nd ^m pair, bearing dispersed W-specific sites, along with structural rearrangement of the pair (pericentric inversion), size heteromorphism, and suppression of recombination during meiosis (Figure 4, step 2). This situation is found in the ZW pair of the *C. lauroi *population analyzed in the present study. Supporting this hypothesis, other populations of *C. lauroi *also presented NOR-bearing Z and W chromosomes and showed a higher differentiation stage of sex chromosomes because of heterochromatin amplification [4]. Afterwards, gene erosion and heterochromatin amplification of the W chromosome would be more accentuated, leading to an accumulation of W-specific sequences associated with the differentiation of morphological types of Z and W chromosomes reported among species/populations of *Characidium*. Such a stage can be visualized in the intermediary developmental stages in *C. lanei *[9,17], or more advanced stages, such as those as in the population of *C. gomesi *from the Alambari stream (Figure 4, step 3).

Simultaneously with the intense W heterochromatin amplification, such as observed among populations of *C. gomesi *(Grande River, Minhoca stream, Verde River) and *C. alipioi*, new translocation events placed the NORs on an autosomal sm pair (17^th ^or 18^th^) (Figure 4, step 4). The maintenance of NORs on sex chromosomes in the population of *C. gomesi *(Alambari stream) strongly suggests that NORs have undergone a novel transposition in other populations, moving to an autosomal pair. This condition could be selectively favored by avoiding the W heterochromatin amplification of some populations, again reinforcing the common origin of the ZW system in *Characidium*.

Even though NORs proved to be highly mobile in the genomes of *Characidium*, most species present a single NOR-bearing pair. Likewise, *C. zebra*, placed as basal in *Characidium *phylogeny [12] shows a single NOR pair. Only two populations of *C. gomesi *from Southern Brazil were divergent, one from the Quebra Perna stream (PR) bearing 10 ribosomal sites [2], and another from the Verde River (PR) bearing six autosomal NORs (present study), clearly representing derived and rare conditions in this genus.

### Biogeoghrapical features and interspecific divergence

The evolutionary history and the chromosomal diversification of *Characidium *are closely related to the isolation of populations in headwaters of distinct hydrographic systems [2,4,5,7]. The main rivers in South and Southeaster Brazil are currently separated by barriers to the dispersal of species and populations, favoring isolation and gene flow restraints [22]. Thus, the populations were adapted to a particular environmental subset, and the occurrence of small-sized species of limited spatial dispersal favor the fixation of interpopulation genetic divergences [1]. Therefore, the karyotype structure and differences in both morphology and composition of sex chromosomes in *Characidium *could have been fixed from the common ancestor of different species/populations (demonstrated in Figure 4), promoting inter- and intraspecific variation and contributing to the speciation process.

## Conclusions

Comparative chromosomal painting using W-specific and 18S rDNA probes in species/populations of *Characidium*, allowed us to infer the differentiation of sex and NOR-bearing chromosomes in this group, gaining a better understanding of the chromosomal and biogeographic features acting on the population differentiation, reproductive isolation, and speciation of this group.

## Methods

### Analyzed species and chromosomal preparations

Chromosomal preparations of *C. zebra*, *C. lauroi*, and *C. gomesi *were analyzed (Table 2). Voucher specimens were deposited at the fish collection of the *Museu Nacional *(MNRJ), Rio de Janeiro, under catalog numbers (MNRJ 22212, 29185) and *Laboratório de Biologia e Genética de Peixes *at UNESP in Botucatu, São Paulo, Brazil, under the numbers (LBP 6733 and 8709).

**Table 2:** Species/populations of the *Characidium *analysed, sampled localities/state, hydrographic basin, number of females (F) and males (M).

Species	Localities (State)	Hydrographic basin	F	M	Total
*C. zebra*	Passa Cinco River (SP)	Alto Paraná	4	8	12
*C. lauroi*	Grande Stream (SP)	Paraíba do Sul	2	3	5
*C. gomesi*	Alambari Stream (SP)	Alto Paraná	22	8	30
*C. gomesi*	Grande River (SP)	Alto Paraná	7	10	17
*C. gomesi*	Minhoca Stream (MG)	São Francisco	5	2	7
*C. gomesi*	Verde River (PR)	Alto Paraná	36	8	44

The chromosomal preparations were obtained from anterior kidney cells by an air-drying technique [23]. The metaphase chromosomes were conventionally stained and C-banded [24] to determine diploid number, chromosome formula, and heteromorphism of the sex chromosomes prior to further analyses using fluorescence *in situ *hybridization (FISH). The procedures were performed in compliance with the Ethics Committee on Animal Experimentation (process number: 04741/08) of the Universidade Estadual de Ponta Grossa (Brazil).

### Chromosomal probes

Two probes were used to map regions/chromosomes in *Characidium *species: (1) An 18S rDNA probe isolated from the total DNA of *Prochilodus argenteus *[25] and; (2) a W-specific probe of *C. gomesi *(population from Minhoca stream, São Francisco River basin) isolated in this study by microdissection of C-banded chromosomes [17], here named the W probe.

### Microdissection of the W chromosome in *Characidium gomesi* and DOP-PCR

The W chromosomes of *C. gomesi *from Minhoca stream, São Francisco River basin, (MG) were microdissected from C-banded metaphase chromosomes to allow precise identification of sex chromosomes. The chromosomal microdissections were carried out using an inverted microscope IX51 (Olympus^®^) equipped with a mechanical micromanipulator (Narishige^®^). Glass capillaries of about 0.7 μm in diameter were prepared using a micropipette puller (Narishige^®^) and were used in the microdissection. Sixteen C-banded W chromosomes of *C. gomesi *were microdissected. The chromosomes were then transferred to a microtube and used in (DOP-PCR). The DOP-PCR amplification [26] and the changes procedures to C-banded chromosomes [17] were used to obtain the W-probe. The DOP-PCR of *C. gomesi *W chromosome consists of 1 × ThermoSequenase reaction buffer, 40 μM dNTPs and 2 μM DOP primer (5' ccg act cga gnn nnn nat gtg g 3'). The microtube was heated to 95°C for 10 min, followed by the addition of 10 U of ThermoSequenase enzyme. The first amplification is carried out through RAMP-PCR: 5 min 94°C; 12 cycles of low stringency (94°C 1 min and 30 s, 32°C 2 min, increasing 0.2°C/s until reaching 72°C and 72°C 2 min); followed by 35 cycle of high stringency (94°C 1 min and 30 s, 52°C 1 min and 30 s and 72°C 1 min and 30 s).

### Fluorescence *in situ* hybridization (FISH)

FISH experiments were carried out in *Characidium *representatives using 18S rDNA and W-chromosome probes. The 18S rDNA probe was labeled by PCR using 16-dUTP-biotin (Roche^®^). In this probe, the PCR mix comprised 20 ng template, 1× *Taq *polymerase buffer (2 mM MgCl_2_), 40 μM of dATP, dGTP and dCTP, 28 μM of dTTP, 12 μM of 16-dUTP biotin, 2 μM of each M13 primer and 0,05 U/μL of *Taq *polymerase. The W probe was labeled by DOP-PCR using 11-dUTP digoxigenin (Roche^®^). The PCR mix comprised 1x *Taq *polymerase buffer (2 mM MgCl_2_), 40 μM of dATP, dGTP and dCTP, 28 μM of dTTP, 12 μM of 11-dUTP digoxigenin, 2 μM of DOP primer, and 0,05 U/μL of *Taq *polymerase under the following conditions: (1×) 94°C 5 min; (35×) 90°C 1 min and 30 s, 52°C 1 min and 30 s, 72°C 1 min and 30 s, and (1×) 72°C 5 min. The FISH procedure [27] was performed under high stringency conditions (2.5 ng/μL of each probe, 50% formamide, 2 × SSC, 10% dextran sulfate). Signal detection was accomplished using AlexaFluor-Streptavidin 488 (Molecular Probes^®^) and antidigoxigenin-Rhodamine (Roche^®^) antibodies. The chromosomes were counterstained with 4',6'-diamino-2-phenylindole (DAPI-0.2 μg/mL) in Vectashield mounting medium (Vector^®^), and analyzed using an Olympus BX41 epifluorescence microscope equipped with the DP 71 image capture system (Olympus^®^). The chromosomes were organized into metacentric (m), submetacentric (sm), and subtelocentric (st), according to arm ratio, and arranged in decreasing order in the karyotype [28].

## List of abbreviations

bp: base pairs; DAPI: 4',6'-diamino-2-phenylindole; DOP-PCR: Degenerate Oligonucleotide primed-Polymerase Chain Reaction; FISH: fluorescence *in situ *hybridization; m: metacentric; MG: Minas Gerais State; NOR: nucleolus organizer region; PR: Paraná State; sm: submetacentric; SP: São Paulo State; st: subtelocentric

## Competing interests

The authors declare that they have no competing interests.

## Authors' contributions

TCM, JCP and MBPA collected the samples, collaborated on all cytogenetic procedures, undertook the bibliographic review, and coordinated the writing of this paper. VN and MCA participated in developing the laboratory techniques, cytogenetic analyses and writing. CO, FF, LACB, OMF and RFA participated in the design of the study, in the inter institution integration study and drafted the manuscript. MRV coordinated the study, helped develop the laboratory techniques, performed specific W-probe of *Characidium*, and reviewed the manuscript. All authors read and approved the final manuscript.

## References

[B1] Buckup PA Reis RE, Kullander SO, Ferraris CJ Jr Family Crenuchidae (South American Darters) Checklist of the freshwater fish of South and Central America 2003 Porto Alegre: Edipucrs 87 95

[B2] Vicari MR Artoni RF Moreira-Filho O Bertollo LAC Diversification of a ZZ/ZW sex chromosome system in *Characidium *fish (Crenuchidae, Characiformes) Genetica 2008 134 311 317 10.1007/s10709-007-9238-2 18175199

[B3] Maistro EL Mata EP Oliveira C Foresti F Unusual occurrence of a ZZ/ZW sex-chromosome system and supernumerary chromosomes in *Characidium cf. fasciatum *(Pisces, Characiformes, Characidiinae) Genetica 1998 104 1 7 10.1023/A:1003242020259 16220371

[B4] Pansonato-Alves JCP Paiva LRS Oliveira C Foresti F Interspecific chromosomal divergences in the genus *Characidium *(Teleostei: Characiformes: Crenuchidae) Neotrop Ichthyol 2010 8 77 86 10.1590/S1679-62252010000100010

[B5] Pansonato-Alves JCP Vicari MR Oliveira C Foresti F Chromosomal diversification in populations of *Characidium cf. gomesi *(Teleostei, Crenuchidae) J Fish Biol 2011 78 183 194 10.1111/j.1095-8649.2010.02847.x 21235554

[B6] Centofante L Bertollo LAC Moreira-Filho O Comparative cytogenetics among sympatric species of *Characidium *(Pisces, Characiformes): Diversity analysis with the description of a ZW sex chromosome system and natural triploidy Caryologia 2001 5 253 260

[B7] Centofante L Bertollo LAC Buckup PA Moreira Filho O Chromosomal divergence and maintenance of sympatric *Characidium *fish species (Crenuchidae, Characidiinae) Hereditas 2003 138 213 218 10.1034/j.1601-5223.2003.01714.x 14641486

[B8] Maistro EL Jesus CM Oliveira C Moreira Filho O Foresti F Cytogenetic analysis of A, -B chromosomes and ZZ/ZW sex chromosomes of *Characidium gomesi *(Teleostei, Characiformes, Crenuchidae) Cytologia 2004 69 181 186 10.1508/cytologia.69.181

[B9] Noleto RB Amorin AP Vicari MR Artoni RF Cestari MM An unusual ZZ/ZW sex chromosome system in *Characidium *fishes (Crenuchidae, Characiformes) with the presence of rDNA sites J Fish Biol 2009 75 448 453 10.1111/j.1095-8649.2009.02342.x 20738550

[B10] Pansonato-Alves JCP Oliveira C Foresti F Karyotypic conservatism in samples of *Characidium cf*. *zebra *(Teleostei, Characiformes, Crenuchidae): Physical mapping of ribosomal genes and natural triploidy Genet Mol Biol 2011 34 208 213 10.1590/S1415-47572011005000005 21734818 PMC3115311

[B11] Miyazawa CS Galetti PM Jr First cytogenetical studies in *Characidium *species (Pisces: Characiformes, Characidiinae) Cytologia 1994 59 73 79

[B12] Buckup PA The monophyly of the Characidiinae, a Neotropical group of characiform fishes (Teleostei: Ostariophysi) Zool J Linn Soc 1993 108 224 245

[B13] Oliveira C Foresti F Almeida-Toledo LF Pisano E, Ozouf-Costaz C, Foresti F, Kapoor BG Karyotypic evolution in Neotropical fishes Fish Cytogenetics 2007 Enfield: Science Publisher 111 164

[B14] Steinemann S Steinemann M Biased distribution of repetitive elements: a landmark for neo-Y chromosome evolution in *Drosophila miranda* Cytogenet Cell Genet 2001 93 228 233 10.1159/000056988 11528116

[B15] Steinemann S Steinemann M Retroelements: tools for sex chromosome Evolution Cytogenet Genome Res 2005 110 134 143 10.1159/000084945 16093665

[B16] Silva DS Milhomem SSR Pieczarka JC Nagamachi CY Cytogenetic studies in *Eigenmannia virescens *(Sternopygidae, Gymnotiformes) and new inferences on the origin of sex chromosomes in the *Eigenmannia *genus BMC Genetics 2009 10 74 19930594 10.1186/1471-2156-10-74 PMC2789750

[B17] Vicari MR Nogaroto V Noleto RB Cestari MM Cioffi MB Almeida MC Moreira-Filho O Bertollo LAC Artoni RF Satellite DNA and chromosomes in Neotropical fishes: Methods, applications and perspectives J Fish Biol 2010 76 1094 1116 10.1111/j.1095-8649.2010.02564.x 20409164

[B18] Reed KM Phillips RB Polymorphism of the nucleolus organizer region (NOR) on the putative sex chromosomes of Arctic char (*Salvelinus alpinus*) is not sex related Chromosoma 1997 5 221 227 10.1023/A:1018411417816 9244448

[B19] Charlesworth D Charlesworth B Marais G Steps in the evolution of heteromorphic sex chromosomes Heredity 2005 95 118 128 10.1038/sj.hdy.6800697 15931241

[B20] Beçak W Beçak ML Cytotaxonomy and chromosomal evolution in Serpents Cytogenetics 1969 8 247 262 10.1159/000130037 5381335

[B21] Singh L Purdom IF Jones KW Sex chromosome associated satellite DNA: evolution and conservation Chromosome Res 1980 79 137 157 10.1007/BF01175181 7398495

[B22] Weitzman SH Menezes NA Weitzman MJ Vanzolini PE, Heyer WR Phylogenetic biogeography of the Glandulocaudini (Teleostei, Characiformes, Characidae) with comments on the distributions of other freshwater fishes in Eastern and Southeastern Brazil Proceedings of a Workshop on Neotropical Distribution Patterns 1988 Rio de Janeiro: Academia Brasileira de Ciências 379 427

[B23] Bertollo LAC Takahash CS Moreira-Filho O Cytotaxonomic considerations on *Hoplias lacerdae *(Pisces, Erythrinidae) Braz J Genet 1978 1 103 120

[B24] Sumner AT A simple technique for demonstrating centromeric heterochromatin Exp Cell Res 1972 75 304 306 10.1016/0014-4827(72)90558-7 4117921

[B25] Hatanaka T Galetti PM Jr Mapping of the 18S and 5S ribosomal RNA genes in the fish *Prochilodus argenteus *Agassiz, 1829 (Characiformes, Prochilodontidae) Genetica 2004 122 239 244 10.1007/s10709-004-2039-y 15609546

[B26] Telenius H Carter NP Bebb CE Nordenskjold M Ponder BA Tunnacliffe A Degenerate oligonucleotide primed PCR: general amplification of target DNA by a single degenerate primer Genomics 1992 13 718 725 10.1016/0888-7543(92)90147-K 1639399

[B27] Pinkel D Straume T Gray JW Cytogenetic analysis using quantitative, high-sensitivity, fluorescence hybridization Proc Natl Acad Sci USA 1986 83 2934 2938 10.1073/pnas.83.9.2934 3458254 PMC323421

[B28] Levan A Fredga K Sandberg AA Nomenclature for centromeric position on chromosomes Hereditas 1964 52 201 220

